# Zinci Oxychloridum

**Published:** 1874-05

**Authors:** John Fairbank


### ARTICLE VII.

### *Zinci Oxychloridum—Oxychloride of Zinc.*

BY JOHN FAIRBANK, ESQ., M. R. C. S.

The points to be observed by the dentist in preparing the oxychloride are—1st. To use the least possible amount of the chloride of zinc solution, in order that the oxychloride may contain the largest possible quantity of oxide. 2nd. To be careful to obtain a perfect mixture of the two salts.

The tendency of all oxychlorides to dissolve and wear away in the mouth has always engaged the attention of the profession, and various means have been devised for remedying the defect. Crystalline Silicates, such as finely powdered glass and clay, have been used to act mechanically as a protection against the chemical and mechanical influences always at work in the mouth, but with only partial success. Mr. Fletcher has, however, lately devised a new mode of protection. By a very careful process he endeavors to coat each grain of the oxide with a varnish which is capable of resisting the action of mineral acids; but unfortunately in this case the means which he takes for protecting the grains of the oxide of zinc from the acids of the mouth must also tend to prevent its perfect combination with the zinc chloride, and this conclusion is borne out by the extreme difficulty one experiences in getting the two to combine, and to form a smooth homogeneous paste; and when one does succeed in obtaining this result, it is only after the grains have been so broken up by the spatula as to destroy the good effect that their coating was to have produced. Mr. Fletcher contends, however, and perhaps with justice, that when mixed with proper care the grains of zinc oxide are not broken up, but only split in half, and that thus only one surface, which is amply sufficient for the absorption the chloride of zinc, is exposed, while the other surfaces remain covered by this coating.

Before mentioning the various cases in which oxychloride might be employed with advantage as a filling, I should like to utter a warning note for the benefit of those who begin to use this filling for their patients good, and end by using it simply for their own convenience and to save themselves trouble. They are in a hurry; the cavity is difficult to get at; a gold filling may not succeed; the tooth is tender too, and the patient does not like pain, so a plastic filling is introduced. Such get into the habit of using it for all difficult cavities, and reserve the gold for the simpler ones; and thus our skill in gold filling never attains perfection, and we have as a consequence many of our countrymen and women actually refusing to consult any but American dentists.

I should like to offer, as an example for us all to follow, that of one of our leading dentists, who, having twice filled a tooth with gold unsuccessfully, still persevered a third time, almost in opposition to his patient's wishes, and finally succeeded in placing a good gold filling in a very difficult cavity. No doubt he lost in a pecuniary sense, for patients were always waiting his pleasure, but the gain to the profession was worth more than the loss of fees.

Osteoplastic fillings may be legitimately used for cavities of large size with exceedingly papery high walls, where it is not advisable to use amalgam, and where you do not wish to cut the wall down; also for cavities where only one high wall remains, the others having broken away; an example of this you frequently get in bicuspid teeth. Also to fill tender and sensitive milk teeth, where you have no reason to suppose that the pulp is seriously affected. It may be used to fill up the lower portion of very deep cavities, such as you sometimes get on the masticating surfaces of molar teeth; in these cases the gold is economized and the pulp protected. But the two most important instances in which the oxychloride may be used are for capping exposed, or very thinly covered pulps; and for filling the pulp cavity and canals of dead teeth.

Oxychloride of zinc should never be used in proximal or buccal cavities, if it is possible to avoid it; neither should it be employed in those cases where the inflammation of the pulp has gone on to suppuration, or to partial or complete devitalization; in such cases the pulp chamber should be at once opened, arsenious acid applied, and the pulp extracted; and I here allow myself to remark that in my own experience, where I have hesitated to adopt this summary mode of treatment, and have tried to preserve the pulp, to save the patient and myself the inconvenience and pain of extirpating it, I have always found it to be the exception when success has attended my efforts; and even in the successful cases, when periods of extreme depression of vital powers have occurred, such as the later periods of gestation, the period of suckling, and after exhausting diseases, that particular tooth has begun to give trouble, whereas, in those cases where the pulp is removed and the pulp chamber and canals well filled any result short of a complete success is almost impossible.—*British Journal of Dental Science.*

---

